# Integration of a 4-gene risk score model enhances prognostic accuracy in acute myeloid leukemia

**DOI:** 10.1080/15384101.2025.2578389

**Published:** 2025-10-29

**Authors:** Yiqing Li, Boqi Li, Jie Xiao, Kezhi Huang, Yuchan Liang, Peiting Zeng, Wenjuan Yang, Danian Nie

**Affiliations:** a Department of Hematology, Sun Yat-Sen Memorial Hospital, Sun Yat-Sen University, Guangzhou, China; b Guangdong Provincial Key Laboratory of Malignant Tumor Epigenetics and Gene Regulation, Sun Yat-sen Memorial Hospital, Sun Yat-sen University, Guangzhou, China; c Department of Hematology, the Sixth Affiliated Hospital, School of Medicine, South China University of Technology, Foshan, China

**Keywords:** Acute myeloid leukemia, bioinformatics, relapse, risk score, European LeukemiaNet

## Abstract

The clinical outcomes of acute myeloid leukemia (AML) patients exhibit substantial heterogeneity, with relapse posing a formidable challenge. Herein, we developed a risk score model by integrating relapse-related genes through Cox regression analysis. The relapse-related genes were identified via differential gene expression analysis of 15 matched diagnosed and relapsed AML samples retrieved from the Gene Expression Omnibus (GEO) database. These genes include *SCN9A*, *CFH*, *CD34*, and *CALCRL*. Our findings demonstrate that higher risk scores were significantly associated with an unfavorable ELN2017 risk classification, leukemic transformation, as well as *FLT3*-ITD and *RUNX1* mutations. Conversely, lower risk scores were linked to *NPM1* mutation. Patients with higher risk scores had a shorter overall survival (OS). Furthermore, we integrated the risk score model with the European LeukemiaNet (ELN) risk classification to establish a novel composite risk classification scheme. Patients were classified into three new risk groups based on composite risk classification showing significantly distinct OS. In summary, the four-gene risk score holds promise in predicting the OS of AML patients, and the composite risk classification shows greater potential in predicting the outcomes of AML patients. These four genes may represent potential therapeutic targets in the treatment of AML.

## Introduction

Acute myeloid leukemia (AML) is a group of heterogeneous hematological diseases and represents the most common type of leukemia in adults. Despite the fact that the prognosis of AML has improved in recent decades with advances in chemotherapy, hematopoietic stem cell transplantation (HSCT), supportive care and new drug discovery, the overall survival (OS) remains unsatisfactory. Statistical results from 2015 to 2021 in the United States showed that the 5-y survival rate of AML patients was 32.9%, while that of elderly patients older than 65 y was only 12.4% [1]. Relapse stands as one of the main causes leading to death among AML patients. Nearly one-third of AML patients fail to achieve complete remission (CR), and even those who do are at significant risk for relapse, thereby reducing OS [2,3]. Early identification of individuals prone to relapse serves as an effective strategy for improving AML outcomes.

At present, there exists a clear consensus on numerous molecular abnormalities associated with AML prognosis such as *NPM1* and *FLT3* mutations [4,5]; thus, identifying novel prognostic genes has become pivotal in determining risk factors for AML. As gene detection techniques continue advancing, more prognostic genes related to AML will be identified and incorporated into guidelines. Several studies have developed prognostic models utilizing gene expression profiling to stratify AML patients with different prognoses.

The DNA microarray technology can be utilized to determine the variation in gene expression between pre- and post-treatment, as well as evaluate the disease prognosis [6,7]. R programming language is one of the most widely used tools for analyzing data derived from microarray gene expression. It offers a diverse range of robust packages and functions that cater to the objective of identifying differentially expressed genes (DEGs) and integrating data from multiple datasets for meta-analysis.

In this study, we retrieved two independent cDNA microarray datasets containing matched diagnosis-relapsed adult AML from the Gene Expression Omnibus (GEO) database [8,9], gene expression profiling was compared between the diagnosed and relapsed AML samples to identify relapse-related genes, then we screened four genes associated to OS through Kaplan – Meier analysis and developed a 4-gene prognostic risk score through Cox proportional hazard model by using the expression and clinical data of 403 AML patients from the Oregon Health and Science University (OHSU) dataset. The prognostic impact of this risk score model was validated in the OHSU dataset as an internal validation cohort, as well as in an external validation cohort from The Cancer Genome Atlas (TCGA) database comprising 172 AML patients. These four genes are anticipated to emerge as novel or previously undiscovered prognostic indicators for AML, thereby providing compelling evidence for improving AML prognosis and identifying novel therapeutic targets.

## Methods

The methods are primarily divided into two parts. The first part involves mining DEGs using GEO datasets. Significantly dysregulated genes are identified by comparing the differences in gene expression between diagnosed and relapsed AML patients. The second part focuses on constructing a risk score model. Genes found to be independently associated with OS in multivariate analysis were further assigned to constructing model. It is important to emphasize that this step aims not to validate all obtained DEGs, but rather to select genes that have been reported as potentially relevant to AML after conducting a literature search among the 30 dysregulated genes exhibiting the most significant differences.

### Selection of microarray datasets

To obtain the matched diagnosis-relapsed AML microarray expression datasets, an extensive search in GEO database (http://www.ncbi.nlm.nih.gov/geo) was performed using the keywords “acute myeloid leukemia OR acute myelogenous leukemia” and selected the study type as Expression profiling by array to present only the results of microarray datasets. The inclusion criteria were as follows: 1) two groups of samples with matched diagnosis-relapsed adult AML; 2) availability of raw data CEL files to make normalization feasible; 3) study type: expression profiling by array; 4) organism: homo sapiens. Two datasets matched with inclusion criteria were screened out from 468 results retrieved before 20th August, 2021, which are GSE66525 [8] and GSE75086 [9]. The former dataset comprises 11 pairs of matched diagnosis-relapsed adult AML samples using the Affymetrix Human Gene 1.1 ST Array, while the latter comprises 11 pairs of matched diagnosis-relapsed adult AML samples using Affymetrix Human Gene 2.0 ST Array. Subsequently, CEL raw files from both datasets were downloaded from GEO database for further analysis.

### Meta-analysis for datasets

The raw data were normalized using the Robust Multichip Averaging function in Oligo package to return log2 transformed intensities [10,11]. The probes were annotated with gene symbols using the Bioconductor biomaRt package, and array probes without gene symbol annotation were filtered out before analysis. In cases where multiple probes targeted the same gene, the probe with the highest average expression value was selected. To integrate microarray data from different platforms, a meta-analysis was performed by RankProd. After filtering and preprocessing of raw data, we merged the expression matrix of different datasets. Dysregulated genes between relapsed AML and matched samples at initial diagnosis were identified using Rank Product (RP) methods [12,13]. Then, genes with percentage of false prediction (pfp) value <5% were considered as DEGs using the topGene function. Among these DEGs, genes with fold change (FC)> 1 were considered upregulated, while those with FC < 1 were considered downregulated.

### Gene set enrichment analysis

Functional annotation clustering of significantly upregulated (pfp < 0.05, FC > 1) and downregulated (pfp < 0.05, FC < 1) genes lists was performed by using the DAVID (Database for Annotation, Visualization, and Integrated Discovery, https://david.ncifcrf.gov/) online bioinformatic tool [14]. Biological Process of Gene Ontology (GO) analysis was selected to identify the biological processes (BP) in which these genes may collectively participate to identify the BP represented in the gene profile.

### Patients

The clinical data of 451 AML patients were obtained from Tyner’s study (OHSU dataset) [15], including the general clinical features such as the gender, age, race, laboratory tests, 2017 European LeukemiaNet (ELN) risk classification, survival data such as survival event, survival time and causes of death. Additionally, it provided information on chromosome karyotypes, fusion genes and gene mutation status of *FLT3*-ITD, *IDH1*, *KRAS*, *NPM1*, *NRAS*, *TP53*, *RUNX1*, *JAK2*, etc. Patients lacking survival data (*n* = 48) were excluded from the analysis. The corresponding gene expression profiling of remaining 403 patients was obtained through R studio. OHSU dataset was used for verifying the association between seven gene expression and OS, constructing a polygenic risk score model as a training cohort and for performing internal validation. An independent dataset derived from TCGA served as an external validation cohort, containing genomic information on 200 adult AML patients [16]. About 172 patients with complete survival data were included in survival analysis. Additional clinical data include general clinical features, white blood cell count, type of HSCT, cytogenetic information, immunophenotype, etc.

### Construction of polygenic risk score model

Through meta-analysis, thousands of DEGs were obtained. A literature search was performed using PubMed for the top 30 dysregulated genes with the most significant expression differences, the candidate genes were applied to survival analysis using the log-rank test for further validate their potential relationship with OS. Patients in OHSU dataset were divided into high expression and low expression groups based on optimal cutoff value of gene expression determined by the “surv_cutpoint” function of the “survminer” package. The differences in OS between the two groups were compared. Genes significantly associated with OS were included in the multivariate analysis to identify genes independently associated with prognosis, a p-value < 0.1 was considered statistically significant. These genes were then applied to construct a risk score model based on gene expression levels weighted by the regression coefficients (*β* values) derived from the multivariate Cox regression analysis. The risk score formula was established as the sum of each gene’s expression level multiplied by its corresponding *β* value: (expression of gene 1×*β1*)+(expression of gene 2 × *β2*)+ ⋯ +(expression of gene n × *βn*).

### Development of a composite risk classification scheme

ELN2017 is a genetics-based risk classification for AML recommended by an international expert panel on behalf of ELN [17], distinguishing AML patients into three risk groups: favorable, intermediate, and adverse. The refined risk stratification of AML is of critical importance, as treatment strategies are determined based on distinct risk categories. Among these considerations, it is important to identify high-risk patients and refer them to appropriate treatment strategies, such as HSCT, which may significantly improve clinical outcomes. In the OHSU dataset, while the ELN2017 classification effectively isolated a favorable-risk group with superior outcomes, it demonstrated a critical limitation for this core objective: it failed to yield a statistically significant difference in overall survival between the intermediate and adverse risk groups after excluding the favorable-risk patients (Figure S1(a)). This inability to further stratify the non-favorable-risk population limits the resolution of prognostic assessment. To directly address this gap and to evaluate whether our model could provide the necessary discriminatory power for high-risk identification, we therefore developed a composite risk classification scheme by integrating the ELN2017 and risk score model, and further clarified the impact of this new classification scheme on patients’ OS through survival analysis.

### Statistical analysis

In OHSU dataset, the Mann-Whitney U test was used to compare the differences in distribution of the medians or the continuous variables between the two subgroups, such as age, laboratory tests, etc. For categorical variables like gender, ELN2017 risk classification, cytogenetic changes, and gene mutations, either the Chi-square test or Fisher’s exact test was utilized. The Spearman correlation test was used to analyze the correlation between gene expressions. The Cox proportional hazards model was used in multivariate regression analysis. The relationship between single gene expression or risk score and patients’ OS was verified using Kaplan–Meier curves and log-rank tests. All statistical analysis was performed in R studio 1.3 and SPSS 26.0.

## Results

### Identification of relapse-related genes in AML and functional annotation clustering for biological process

**Figure 1.**
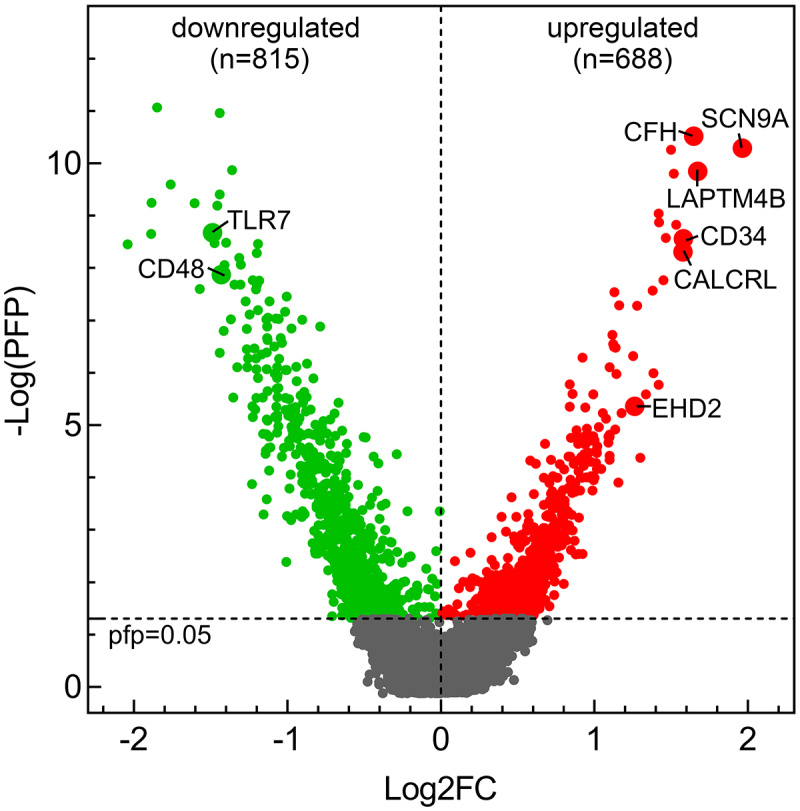
Volcano plot for the comparison between the diagnosed and relapsed adult AML. The cutoff values FC > 1 and pfp < 0.05 were utilized to identify differentially expressed genes. Nonsignificant genes were shown in gray color. Red color indicates upregulated genes and green color indicates downregulated genes. Genes discussed in our study were shown in the figure.

**Figure 2.**
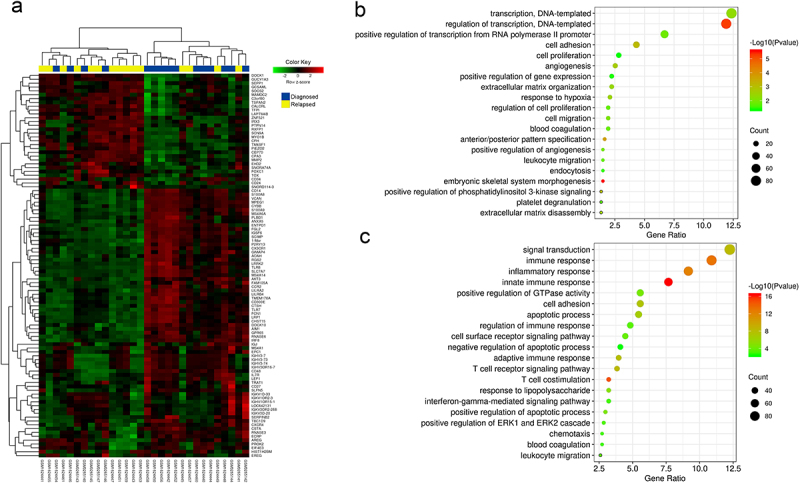
Heatmap of the top 100 DEGs and scatter plot of top 20 enriched BP. (a) Blue color indicates diagnosed AML samples while yellow color indicates relapsed AML samples. Expression levels are represented by red (high expression) and green (low expression). (b) The 20 most significant BP associated with genes upregulated in relapsed AML. (c) The 20 most significant BP associated with genes downregulated in relapsed AML. Gene ratio indicates the DEG count to the total gene count in each BP term. The color of the dots represents the range of the – log10 (P-value), and the size of the dots represents the number of DEGs mapped to the indicated BP term.

After filtering, the final expression matrix consisted of expression values of 22,590 genes from 11 matched diagnosis-relapsed AML samples in GSE66525, and 34,661 genes from four matched samples in GSE75086. The expression values were log2 transformed (Figure S2). Individual analysis of each dataset demonstrated discordant results (Figure S3). To reduce errors due to small sample size and different platforms, the RP.advance function of RankProd was used to perform meta-analysis on the combined expression matrix. This approach integrated quantile-normalized and log-transformed probe intensity signal data from a total of 15 matched diagnosis-relapsed samples across both datasets. The combined matrix comprised a non-redundant set of 20,832 genes. The threshold of percentage of false positives (pfp) ≤0.05 was considered statistically significant. Among these genes, we identified 688 upregulated genes (FC > 1) and 815 downregulated genes (FC < 1) in relapsed adult AML (Table 1 shows the top 30 significantly dysregulated genes). Volcano plot illustrates the distribution of DEGs (Figure 1). The significance of differential expression for the top 100 DEGs was depicted in Figure 2(a) via hierarchical clustering analysis.

**Table 1.** Top 30 significantly dysregulated genes.

Gene symbol	FC	pfp	*p*
**Upregulated genes (*n* = 13)**			
*SCN9A*	3.903	< 0.001	< 0.001
*LAPTM4B*	3.190	< 0.001	< 0.001
*CFH*	3.133	< 0.001	< 0.001
*CD34*	2.990	< 0.001	< 0.001
*CALCRL*	2.981	< 0.001	< 0.001
*CEP70*	2.892	< 0.001	< 0.001
*TM4SF1*	2.863	< 0.001	< 0.001
*ZNF521*	2.826	< 0.001	< 0.001
*CD24*	2.761	< 0.001	< 0.001
*TFPI*	2.732	< 0.001	< 0.001
*IRX3*	2.679	< 0.001	< 0.001
*GUCY1A3*	2.673	< 0.001	< 0.001
*PTPN14*	2.672	< 0.001	< 0.001
**Downregulated genes (*n* = 17)**			
*EPC1*	0.243	< 0.001	< 0.001
*IGHV3-7*	0.270	< 0.001	< 0.001
*AIM1*	0.270	< 0.001	< 0.001
*MPEG1*	0.277	< 0.001	< 0.001
*CYBB*	0.295	< 0.001	< 0.001
*MS4A6A*	0.329	< 0.001	< 0.001
*RNASE6*	0.336	< 0.001	< 0.001
*TLR7*	0.357	< 0.001	< 0.001
*EIF4E3*	0.360	< 0.001	< 0.001
*SCIMP*	0.364	< 0.001	< 0.001
*RNASE3*	0.368	< 0.001	< 0.001
*FGL2*	0.368	< 0.001	< 0.001
*VCAN*	0.368	< 0.001	< 0.001
*CD48*	0.371	< 0.001	< 0.001
*IGHV3-74*	0.375	< 0.001	< 0.001
*PROK2*	0.376	< 0.001	< 0.001
*PLBD1*	0.379	< 0.001	< 0.001

*p* < 0.05 is considered statistically significant.

FC, fold change, [relapsed]/[diagnosed]; pfp, percentage of false prediction.

The DAVID tool was used for functional annotation clustering of gene biological processes. As depicted in Figure 2(b,c), the top three upregulated BP were “transcription, DNA-templated,” “regulation of transcription, DNA-templated,” and “positive regulation of transcription from RNA polymerase II promoter,” whereas the top three downregulated processes were “signal transduction,” “immune response,” and “inflammatory response.”

### Construction of a 4-gene risk score model

**Figure 3.**
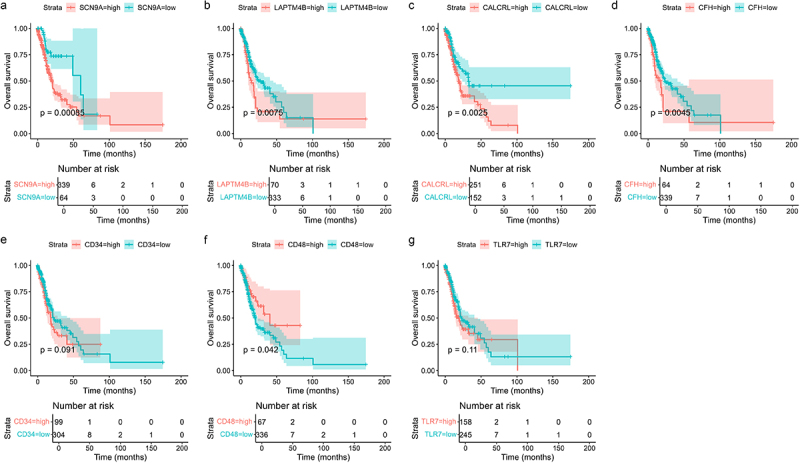
Kaplan-Meier analysis of overall survival stratified by single gene expression level. (a) *SCN9A*, (b) *LAPTM4B*, (c) *CALCRL*, (d) *CFH*, (e) *CD34*, (f) *CD48*, (g) *TLR7*. *p* < 0.1 is considered statistically significant. Genes with statistical significance were further assigned to multivariate cox regression analysis.

Through a literature search among the top 30 dysregulated genes, *SCN9A*, *LAPTM4B*, *CALCRL*, *CFH*, *CD34*, *CD48*, and *TLR7* were found to exhibit a potential relationship with the prognosis of AML. In order to verify the predictive ability, Kaplan – Meier survival analysis was performed on the expression levels of these seven genes. The results revealed that six genes were significantly associated with OS in patients (*p* < 0.1), including *SCN9A* (*p* = 0.00085), *LAPTM4B* (*p* = 0.0075), *CFH* (*p* = 0.0048), *CD34* (*p* = 0.091), *CALCRL* (*p* = 0.0025) and *CD48* (*p* = 0.0025). The high expression of the first five genes, which were found to be upregulated, and the low expression of the last gene, which was found to be downregulated in relapsed AML patients, were related to poor OS (Figure 3). Then, we performed multivariate Cox regression analysis on these six genes to identify independent predictors of OS. After two rounds of analysis, *SCN9A*, *CFH*, *CD34*, and *CALCRL* were found to be statistically significant (Table S1–2). The expression levels and corresponding β values of these four genes were used to calculate the risk score. The final formula is as follows: risk score = 0.81×[*SCN9A*]+0.43×[*CFH*]+0.42×[*CALCRL*]+0.38×[*CD34*].

### Comparison of clinical characteristics, gene mutation and fusion gene

The 403 patients in training cohort were divided into high-risk score group (*n* = 297) and low-risk score group (*n* = 106) based on the optimal cutoff value of 4-gene risk score determined by “surv_cutpoint” function in R studio. As shown in Table 2, compared with low-risk score group, patients in high-risk score group exhibited significantly lower platelet counts (*p* = 0.009) and tended to be male (*p =* 0.017). Furthermore, patients with high risk scores were more frequently categorized into ELN2017 adverse risk subgroups (41.8% vs 18.9%, *p* < 0.001) but less frequently categorized into ELN2017 favorable risk subgroups (19.5% vs 42.5%, *p* = 0.001) compared with those with a low-risk score. As listed in Table 3, high-risk score patients had significantly higher incidences of gene mutations of *FLT3-*ITD (26.6% vs 13.3%, *p* = 0.006), *CEBPA* (17.9% vs 4.8%, *p =* 0.036) and *RUNX1* (49.2% vs 30.0%, *p* = 0.007), and while exhibiting a lower incidence of *NPM1* gene mutation (16.3% vs 48.6%, *p* < 0.001). Moreover, in terms of the incidence of common fusion genes, with the exception of *MLLT3-KMT2A* which exhibited a higher prevalence in the low-risk group (10.8% vs 0.0%, *p* < 0.001) and *CBFB-MYH11* which exhibited a higher prevalence in the high-risk score group (7.7% % vs 1.1%, *p* = 0.038), there were no statistically significant differences observed between the two groups for other fusion genes such as *PML-RARA*, *RUNX1-RUNX1T1*, *GATA-MECOM* (Table S3). We also compared the types of treatments received by patients and found that a greater proportion of patients in the high-risk score group received targeted drugs compared to those in the low-risk score group (21.3% vs 9.7%, *p* = 0.009) (Table S4). The OHSU dataset provides information on whether AML was de novo, relapsed, or secondary to myelodysplastic syndrome (MDS)/myeloproliferative neoplasms (MPN). By conducting chi-square tests to compare differences in incidence rates between high- and low-risk score group for these events, it was determined that there was a higher proportion of patients with prior MDS/MPN in the high-risk score group compared to those in the low-risk score group (18.2% vs 9.4%, *p* = 0.034) (Table 2).

**Table 2.** Comparison of general clinical features between patients in high- and low-risk score subgroup.

Clinical features	Total	Low-risk score (n = 106)	High-risk score (n = 297)	*p*
**Age, median, y**	57	57	56	0.669
**Sex, n (%)**				0.017
Male	226 (56.1)	49 (46.2)	177 (59.6)	
Female	177 (43.9)	57 (53.8)	120 (40.4)	
**Laboratory data, median**				
WBC, × 10^9^/L	37.36	36.18	37.78	0.930
HGB, × g/L	8.7	8.7	8.6	0.978
PLT, × 10^9^/L	66	77	63	0.009
Blasts in BM, %	59.2	58.7	59.4	0.749
Blasts in PB, %	45.1	42.9	45.8	0.390
**ELN2017, n (%)**				
Fav	103(25.6)	45 (42.5)	58 (19.5)	< 0.001
Adv	144 (35.7)	20 (18.9)	124 (41.8)	< 0.001
IM	135 (33.5)	34 (32.1)	101 (34.0)	0.718
Fav Or IM	13 (3.2)	5 (4.7)	8 (2.7)	0.489
IM Or Adv	7 (1.7)	1 (0.9)	6 (2.0)	0.768
Unknown	1 (0.3)	1 (0.9)	0 (0)	
**Other events, n (%)**				
De novo	198 (49.1)	58(29.3)	140 (47.1)	0.180
Relapsed	22 (5.5)	3 (2.8)	19 (6.4)	0.165
Transformed*	64 (15.9)	10 (9.4)	54 (18.2)	0.034

*p* < 0.05 is considered statistically significant.

Adv, adverse; BM, bone marrow; Fav, favorable; HGB, hemoglobin; IM, intermediate; NOS, not otherwise specified; PB, peripheral blood; PLT, platelet count; WBC, white blood cell count.

*Number of patients with prior myelodysplastic syndrome or myeloproliferative neoplasms before leukemic transformation.

**Table 3.** Comparison of gene mutations between patients in high- and low-risk score subgroup.

Gene symbol	Number of samples tested*	Number of samples with gene mutation			*p*
		Total	Low-risk score	High-risk score	
*CEBPA*	176	26	2/42 (4.8%)	24/134(17.9%)	0.036
*FLT3*-ITD	402	93	14/105(13.3%)	79/297(26.6%)	0.006
*IDH1*	181	25	10/49(20.4%)	15/132 (11.4%)	0.117
*KRAS*	136	14	5/34(14.7%)	9/102(8.8%)	0.515
*NPM1*	400	99	51/105(48.6%)	48/295(16.3%)	< 0.001
*NRAS*	152	43	11/37(29.7%)	32/115(27.8%)	0.823
*TP53*	135	24	2/32 (6.3%)	22/81(21.4%)	0.051
*JAK2*	113	9	2/28(7.1%)	7/85(8.2%)	1
*KIT*	151	8	0/37(0.0%)	8/114(7.0%)	0.217
*RUNX1*	74	30	1/15(6.7%)	29/59(49.2%)	0.007
*WT1*	67	25	9/21(42.0%)	16/46(34.8%)	0.526
*DNMT3A*	151	56	19/39(48.7%)	37/112 (33.0%)	0.081
*IDH2*	176	33	9/49 (18.4%)	24/127(18.9%)	0.936

*p* < 0.05 is considered statistically significant.

*Total number of patients with each gene mutation status tested are different.

### Relationship between risk score and OS

**Figure 4.**
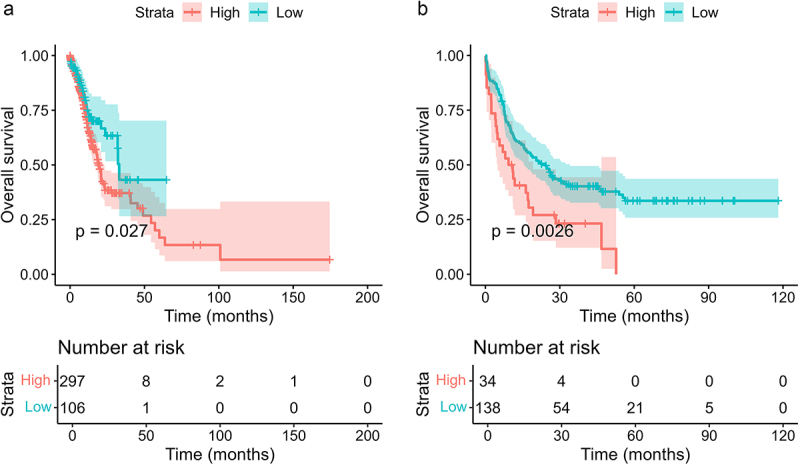
The Kaplan–Meier curves of overall survival stratified by risk score. (a) OHSU cohort. (b) TGCA cohort. Patients in high-risk score groups have shorter OS in both OHSU and TCGA cohort.

Using the optimal cutoff value, we divided 403 patients from the OHSU dataset into high- or low-risk score groups based on their risk scores. The OS of the high-risk score group was significantly worse than that of the low-risk score group in both the OHSU cohort and the TCGA cohort, as depicted in Figure 4(a,b). In the OHSU cohort, the median survival time for the high-risk and low-risk groups was 19.1 months and 33.1 months, respectively (*p* = 0.027). The 2-y survival rates were 38.4% and 63.4%. Similarly, in the TCGA cohort, the median survival time for the high-risk and low-risk groups was 10.2 months and 24.1 months, respectively (*p* = 0.0026), with respective 2-y survival rates of 27.1% and 50.5%. After adjusting for other variables, including age, WBC count, HSCT, and complex karyotype (all of which had p-values < 0.1 in univariate Cox regression analysis) (Table S5), multivariate Cox regression analysis revealed that high risk scores remained significantly associated with worse overall survival among patients in the TCGA cohort (Table 4).

**Table 4.** Multivariate Cox regression analysis of overall survival in TCGA cohort.

Variables	HR	95% Confidence Interval		*p*
		Lower limit	Upper limit	
High risk score	1.732	1.446	2.466	0.016
Elderley ( > 60 y old)	2.814	1.871	4.233	< 0.001
WBC count*	1.008	1.004	1.012	< 0.001
HSCT	0.568	0.375	0.861	0.008
Complex karyotype	2.266	1.287	2.163	0.004

*p* < 0.05 is considered statistically significant.

All the above variables were *p* < 0.1 in univariate analysis.

HR, hazard ratio; WBC, white blood cell; HSCT, hematopoietic stem cell transplantation.

*Continuous variable.

### Development of the composite risk classification scheme

**Figure 5.**
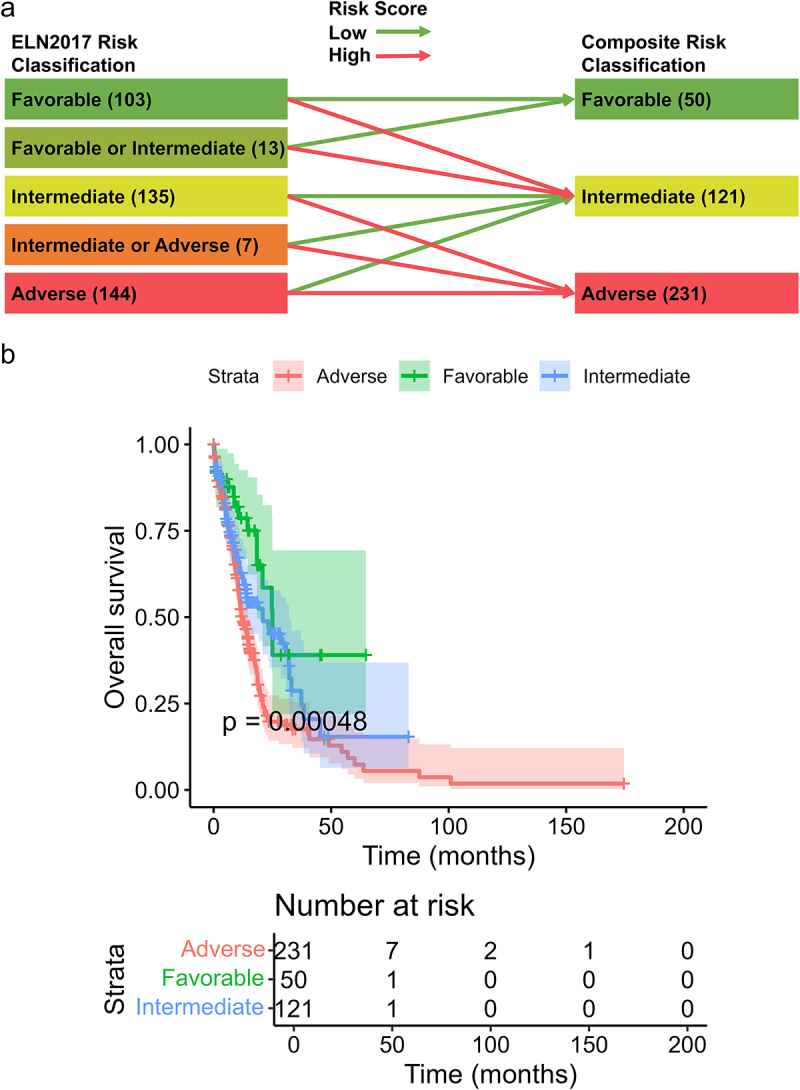
Integration of risk score with ELN2017 risk classification. (a) Scheme of reclassification by integrating the 4-gene risk score with ELN2017 risk classification. (b) The Kaplan–Meier curves of overall survival stratified by three novel integrated risk groups.

The ELN risk classification scheme for adult AML based on abnormal cytogenetic and molecular genetic factors is widely used in clinical practice. Among the 403 patients with survival data in the OHSU cohort, 402 patients were stratified into five risk groups based on the ELN2017 risk classification scheme: “favorable,” “favorable or intermediate,” “intermediate,” “intermediate or adverse,” and “adverse.” The Kaplan–Meier survival curves demonstrated no significant differences in OS among the “intermediate,” “intermediate or adverse,” and “adverse” risk groups (Figure S1 (a)). We integrated ELN2017 with our constructed risk score model to develop a novel composite risk classification scheme (Figure 5(a)). This scheme classified patients as follows: favorable, including ELN2017 favorable risk or “favorable or intermediate” risk patients with low risk scores; intermediate, including ELN2017 favorable risk or “favorable or intermediate” risk patients with high risk scores, as well as ELN2017 intermediate risk, “intermediate or adverse” risk, or adverse risk patients with low risk scores; and adverse, including ELN2017 intermediate risk, “intermediate or adverse” risk, or adverse risk patients with high risk scores. Further survival analysis demonstrated significant differences in OS among these three groups of patients stratified according to this composite scheme, as shown in Figure 5(b) (*p* < 0.001).

**Figure 6.**
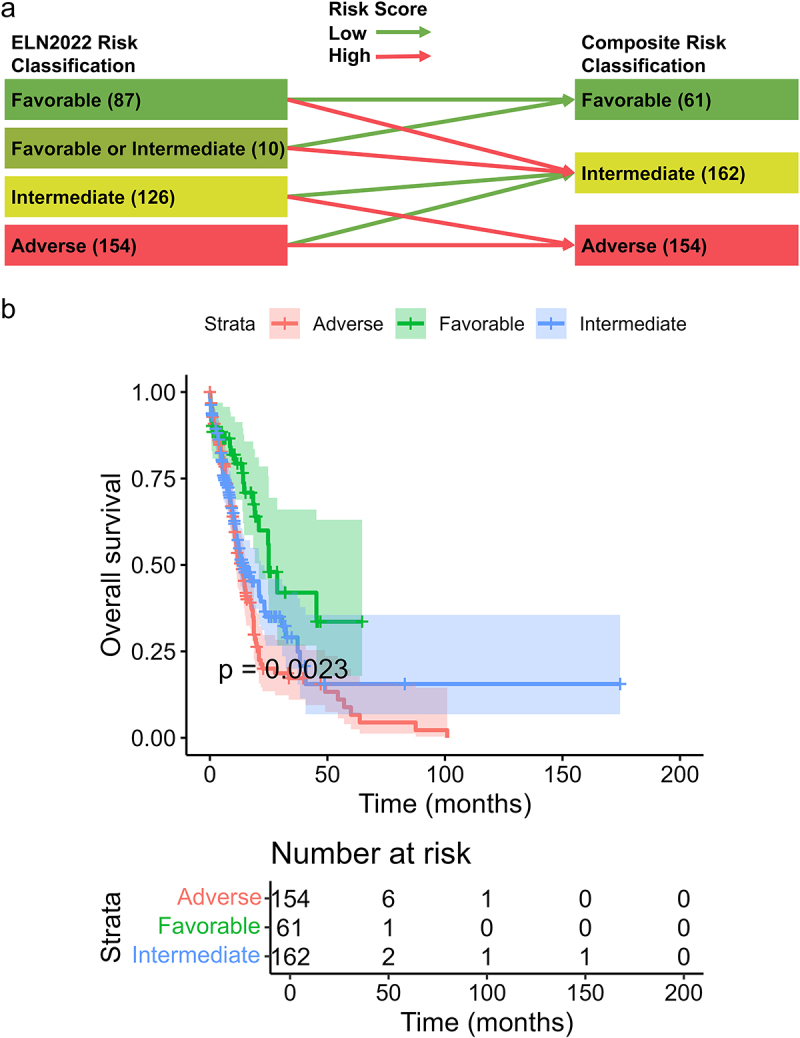
Integration of risk score with ELN2022 risk classification. (a) Scheme of reclassification by integrating the 4-gene risk score with ELN2022 risk classification. (b) The Kaplan–Meier curves of overall survival stratified by three novel integrated risk groups.

Following the widespread recognition of the ELN2017 risk classification, a refined genetic risk classification was introduced with the update to the 2022 edition of recommendations [5]. Given that the original data did not provide information on ELN2022 risk classification, we classified patients in the OHSU cohort based on ELN2022 risk classification for patients with evaluable cytogenetic and molecular genetic test results. A total of 377 patients were divided into four groups: favorable (*n* = 87), favorable or intermediate (*n* = 10), intermediate (*n* = 126), and adverse (*n* = 154). The Kaplan–Meier survival curves demonstrated no significant differences in OS among the “intermediate” and “adverse” risk groups (Figure S1 (b)). As illustrated in Figure 6(a), we developed another composite risk classification scheme by integrating ELN2022 and the risk score model. The survival curves demonstrated significant differences in OS (*p* = 0.0023) (Figure 6(b)).

## Discussion

Accurate prognostic assessment plays a crucial role for researchers and physicians in managing AML because AML is highly heterogeneous and mostly has a poor prognosis. While numerous studies have utilized cDNA microarray technology to investigate the correlation between gene expression profiling and AML prognosis, limited research explores the potential molecular mechanisms underlying poor outcomes by comparing gene expression differences between diagnosed and relapsed AML patients [8,9]. Comparing paired samples from AML patients at initial diagnosis and relapse is particularly valuable for identifying genes highly relevant to AML pathogenesis, relapse, and drug resistance. It also helps to elucidate the underlying molecular mechanisms. However, due to the scarcity of studies focusing on gene expression profiling of matched diagnosed and relapsed adult AML cases, and because most such studies have small sample sizes, the results have varied significantly across studies. In this case, we collected datasets from two distinct studies and performed meta-analysis using robust statistical methods to merge both datasets into a comprehensive analysis aiming to obtain more valuable and convincing insights into genes associated with relapse.

In this study, we aimed to integrate the expression of four relapse-related genes (*SCN9A*, *CFH*, *CD34*, and *CALCRL*) into a prognostic risk score model using the Cox proportional hazards model. Since the relapse of leukemia is a crucial determinant of poor outcome, it is reasonable to assume that the expression profile of these genes has a significant impact on the prognosis of AML patients. To elucidate the relationship between gene expression levels obtained through meta-analysis and survival in AML patients, we developed a risk score model based on these four genes for predicting clinical outcomes. In this model, in both the OHSU and TCGA cohorts, patients with high risk scores exhibited inferior OS compared to those with low risk scores. Multivariate analysis conducted on TCGA cohort further revealed that our risk score could independently predict OS regardless of factors such as WBC count, complex chromosomal karyotype, age, and HSCT. Furthermore, our risk score model was consistent with the ELN2017 risk classification, wherein most patients in the low-risk score group were classified as belonging to the ELN2017 favorable risk group, while those in the high-risk score group were predominantly classified into the ELN2017 adverse risk group. Overall, this further substantiates that the risk score model constructed using meta-analysis-derived DEGs is closely associated with AML patient prognosis.

More importantly, we showed that this 4-gene risk score model may improve ELN risk classification. ELN risk classification is widely accepted and applied by hematologists. The updated ELN2022 guidelines incorporate significant revisions from ELN2017 [5]. However, patients may not accurately be stratified into distinct risk groups even when based on ELN risk classification in clinical practice. Taking the OHSU dataset as an example, among the five groups of patients stratified according to ELN2017, we found that except for the favorable risk group with the best OS, the remaining groups have no significant difference in OS. The median OS across these five groups was as follows: favorable risk group: Not Reached; favorable or intermediate risk group: 14.4 months; intermediate risk group: 20.9 months; intermediate or adverse risk group: 10.8 months; adverse risk group: 18.3 months. Similar observations were made using ELN2022 risk classification (Figure S1). To address the ambiguity present in the OHSU dataset regarding ELN2017 and ELN2022 risk classification, we integrated ELN2017 or ELN2022 with our 4-gene risk score model to develop a novel composite risk classification scheme. The results from survival analysis suggested that the new risk classification appears to hold greater clinical significance than both ELN2017 and ELN2022 in identifying high-risk patients.

Among the four genes used to construct the risk score model, *CD34* is a widely accepted marker of LSCs associated with chemo-resistance and relapse in AML. However, the roles of the other three genes in AML have been less frequently addressed in previous studies. Although these genes may play a role in specific BP, there remains a paucity of reports elucidating their relationship with AML.

The SCN9A gene encodes Nav1.7, a voltage-gated sodium channel and is recognized as an oncogene and has been implicated in tumor initiation, progression, and drug resistance [18–23]. Peptide toxins such as JZTX-I, which activate Nav1.7, can upregulate the expression of cytoskeleton-related proteins and activate the Rho GTPases-RhoA/Rac1 signaling pathway in prostate cancer cells [24]. Marroncini found that low extracellular Na+ could activate the RhoA/ROCK signaling pathway, leading to actin cytoskeleton rearrangements, thereby elevating proliferation, migration, and invasion of chronic myeloid leukemia (CML) cells in vitro [25]. The activation of the RhoA/ROCK pathway has also been demonstrated in AML, where it regulates cell differentiation [26,27]. Another Rho GTPase, Rac1, has been found to enhance the metastatic and proliferative ability of AML; conversely, its absence impairs AML cell survival and growth [28,29]. Furthermore, studies have shown that *FLT3*-ITD and *Rac1* can co-regulate DNA repair, affecting chemotherapy sensitivity in AML cells [30]. However, whether these mechanisms are due to the high expression of Nav1.7 upstream has not been clearly studied. Therefore, further investigation is warranted to determine if SCN9A can affect the proliferation, chemotherapy resistance, and recurrence of AML by regulating sodium channels through a mechanism similar to that observed in solid tumors.

Complement factor H (CFH) is a soluble regulator of the complement system, playing a crucial role in modulating immune tolerance. In our study, *CFH* was significantly upregulated in relapsed AML. Recent studies have confirmed that tumor cells can “hijack” the complement system by overexpressing *CFH*, thereby evading complement attack [31,32]. Isabelle Laverdière et al. [33] found that high expression of *CFH* is closely related to poor prognosis for AML patients under 60 y old and even outperformed *FLT3*/*NPM1* as a prognostic marker for intermediate-risk AML patients. Lee SW [34] reported higher levels of serum CFH in AML patients at diagnosis (PreCR); non-remission patients showed higher levels compared to those who achieved CR. Elevated levels of CFH were also observed in the serum of relapsed patients. Our enrichment analysis results indicate that the downregulated genes are predominantly enriched in the “immune response” biological process. This implies that a dysregulated immune response plays a pivotal role in the pathogenesis and recurrence of leukemia. Although the precise mechanism through which CFH regulates the immune response of leukemia cells is not fully elucidated, we can infer from these studies that *CFH* may serve as a prognostic marker for AML and may even have the potential to become a novel therapeutic target.

The CR rate of AML patients has shown improvement following induction chemotherapy. However, relapse after achieving CR remains a significant factor contributing to the decline in OS. Although new targeted drugs offer hope for AML treatment, drug resistance continues to pose a major challenge. In our study, we found that the fourth gene, CALCRL, is related to drug resistance. *CALCRL* encodes calcitonin receptor-like receptors, including calcitonin gene-related peptide (CGRP) receptor, adrenomedullin (ADM) receptor, and CGRP/ADM dual receptor, which are related to apoptosis regulation, inflammatory response, and cell proliferation [35,36]. Recent studies reveal that *CALCRL* plays a critical role in leukemia. Wagner S showed that high expression values of three genes, including *CALCRL*, could effectively predict response to chemotherapy and the possibility of AML relapse [37]. Sha K et al. [38] also found high *CALCRL* expression was associated with poor survival, early relapse, and chemoresistance in AML. *CALCRL* knockdown or CGRP antagonists effectively reversed drug resistance induced by CGRP, thereby enhancing the effect of cytarabine or daunorubicin in promoting AML cell apoptosis [39,40]. In addition, it has been found in *FLT3*-ITD and *DNMT3A*-R882 double mutant AML cell models that knockdown of *CALCRL* inhibited AML cell proliferation and stemness, and promoted apoptosis of AML cells. Knockdown of *CALCRL* was also found to limit tumor growth and chemotherapy resistance in animal models [41]. Rong Wang et al. [42] also identified high *CALCRL* expression as a poor prognostic factor in *AML*/*ETO*-positive AML patients. Another ADM-CALCRL axis has also been shown to be associated with chemoresistance in AML cells. Larrue C et al. [43] observed that high levels of CALCRL or ADM were associated with reduced CR, 5-y OS, event-free survival, and increased relapse rates in patients. CALCRL can regulate the balance between survival and apoptosis of AML cells by maintaining the stemness of leukemia cells. It also enhances tolerance to chemotherapeutic drugs, promotes cell proliferation, and inhibits apoptosis. For a better understanding of the underlying relationship between *CALCRL* and chemotherapy resistance, the correlation between the expression of *CALCRL* and three ATP-binding cassette family drug resistance-related genes, *ABCB1*, *ABCC1*, and *ABCG2*, was analyzed by Spearman correlation in our study. The results revealed a positive correlation between *CALCRL* expression and the aforementioned drug resistance-related genes (Table S6). Therefore, it can be considered that *CALCRL* represents a potential biomarker and therapeutic target for adult AML. However, specific biological mechanisms remain to be elucidated.

It is important to note that the specific risk-score cutoff is a proof-of-concept value. While it showed consistency in internal validation, its generalizability is limited and must be reestablished for clinical use. Future translation is both necessary and feasible. A logical next step would be to develop a multiplex RT-qPCR assay, which is ideally suited for clinical adoption due to its standardization, low cost, and rapid turnaround time. For this standardized test, a future clinically viable cutoff will not be a direct transfer of the current value, but rather a new value derived from the normalized data (e.g. using the ΔΔCq method) generated by the standardized RT-qPCR assay in a large, prospective, multi-center study. Thus, the primary contribution of this work is the discovery and validation of the risk score model itself, which offers a novel and potent biomarker candidate for AML risk stratification. We provide a foundational framework upon which future translational studies can build to ultimately bring this biomarker into clinical practice.

There are several limitations in this study. The gene expression levels in our collected datasets for differential analysis were measured using microarray technology instead of next-generation sequencing methods known for higher accuracy [44]. Additionally, our meta-analysis combined two GEO datasets with a small total sample size, leading to limited credibility in analysis results. During validation, we observed that the *CBFB-MYH11* fusion gene, a well-established marker of favorable prognosis, was significantly enriched in the high-risk group (Table S3), suggesting underlying prognostic heterogeneity. Multivariate analysis confirmed the independence of our risk score from *CBFB-MYH11* status (Table S7), indicating that the signature captures distinct biological features. This may reflect the influence of other high-risk factors – such as *NRAS* mutations or persistent measurable residual disease – known to adversely impact outcomes in AML with *CBFB-MYH11* [45]. While this finding and its clinical implications require validation in larger, prospective cohorts, it is also important to note that survival analysis on DEGs utilized clinical data from the OHSU dataset. This dataset indicated patient relapse status without specifying times to relapse, thus preventing verification of gene expression’s impact on cumulative relapse rate, despite its established association with relapsed/refractory AML in existing literature. Results of the chi-square test showed that the difference in proportion of relapsed AML between high- and low-risk score subgroups was not significant; this test ignored the times as an important factor of relapse, so the relationship between relapse and risk score cannot be completely determined. More complete clinical data for validation are warranted.

## Conclusions

Based on the fact that relapse is the main factor leading to poor outcome in AML patients, we tried to find new prognostic biomarkers by comparing the differences in gene expression between matched diagnosed and relapsed AML patients obtained from GEO datasets. A total of 1474 genes were identified to be significantly differentially expressed, among which 653 were up-regulated and 821 were down-regulated in relapsed AML. Four genes (*SCN9A*, *CFH*, *CD34*, and *CALCRL*) with prognostic significance were assigned to construct a risk score model. High risk scores were related to the known adverse prognostic factors and shorter OS. These four genes can be used as predictive biomarkers for prognosis of AML, through dynamically detecting the changing trends of their levels to provide a prognostic assessment. These findings may provide a basis for researchers to develop targeted new therapies for AML. In addition, they offer more therapeutic targets for AML patients in the future. Although the model is exploratory, integrating this model with ELN2017 or ELN2022 is anticipated to enhance the precision of AML risk classification.

## Supplementary Material

Supplementary materials.docx

## Data Availability

All the original data used in this study are available in GEO (https://www.ncbi.nlm.nih.gov/geo) and TCGA (https://portal.gdc.cancer.gov/). R scripts for analyzing data are available from the corresponding author upon reasonable request.

## Abbreviations

AML: Acute Myeloid Leukemia
HSCT: Hematopoietic Stem Cell Transplantation
OS: Overall Survival
CR: Complete Remission
DEGs: Differentially Expressed Genes
GEO: Gene Expression Omnibus
OHSU: Oregon Health And Science University
TCGA: The Cancer Genome Atlas
RP: Rank Product
pfp: Percentage of False Prediction
FC: Fold Change
GO: Gene Ontology
BP: Biological Processes
ELN: European LeukemiaNet
MDS: Myelodysplastic Syndrome
MPN: Myeloproliferative Neoplasms
CML: Chronic Myeloid Leukemia
CGRP: Calcitonin Gene-Related Peptides
