# Supplementary material for: Integration of a 4-gene risk score model enhances prognostic accuracy in acute myeloid leukemia
Source: Cell Cycle. 2025 Oct 29;24(21-24):638–54. doi: 10.1080/15384101.2025.2578389 (PMC12710893; doi:10.1080/15384101.2025.2578389)
Supplement: Supplementary materials.docx [file KCCY_A_2578389_SM8818.docx]

**Table S1** Summary of results of first multivariate Cox proportional hazards regression analysis from R studio

|  | **coef** | **exp(coef)** | **se(coef)** | **z** | **Pr(>\|z\|)** | **exp(-coef)** | **lower .95** | **upper .95** |
| --- | --- | --- | --- | --- | --- | --- | --- | --- |
| *SCN9A*low | -0.79285 | 0.45255 | 0.28068 | -2.825 | 0.00473 | 2.2097 | 0.2611 | 0.7845 |
| *CFH*low | -0.38199 | 0.68250 | 0.21882 | -1.746 | 0.08087 | 1.4652 | 0.4445 | 1.0480 |
| *CD34*low | -0.35294 | 0.70262 | 0.18453 | -1.913 | 0.05580 | 1.4233 | 0.4894 | 1.0088 |
| *CALCRL*low | -0.39478 | 0.67383 | 0.18544 | -2.129 | 0.03326 | 1.4841 | 0.4685 | 0.9692 |
| *CD48*low | 0.31395 | 1.36882 | 0.25195 | 1.246 | 0.21275 | 0.7306 | 0.8354 | 2.2429 |
| *LAPTM4B*low | -0.01554 | 0.98458 | 0.21551 | -0.072 | 0.94252 | 1.0157 | 0.6454 | 1.5021 |

coef: coefficients, beta value.

exp(coef): hazard ratio.

se(coef): standard error.

z: Wald value.

Pr(>|z|): *p* value.

**Table S2** Summary of results of second multivariate Cox proportional hazards regression analysis from R studio

|  | **coef** | **exp(coef)** | **se(coef)** | **z** | **Pr(>\|z\|)** | **exp(-coef)** | **lower .95** | **upper .95** |
| --- | --- | --- | --- | --- | --- | --- | --- | --- |
| *SCN9A*low | -0.8059 | 0.4467 | 0.2778 | -2.901 | 0.00372 | 2.239 | 0.2591 | 0.7700 |
| *CFH*low | -0.4302 | 0.6504 | 0.2018 | -2.132 | 0.03301 | 1.538 | 0.4379 | 0.9659 |
| *CALCRL*low | -0.4176 | 0.6586 | 0.1829 | -2.284 | 0.02240 | 1.518 | 0.4602 | 0.9425 |
| *CD34*low | -0.3759 | 0.6867 | 0.1810 | -2.076 | 0.03787 | 1.456 | 0.4816 | 0.9792 |

coef: coefficients, beta value.

exp(coef): hazard ratio.

se(coef): standard error.

z: Wald value.

Pr(>|z|): *p* value.

**Table S3** Comparison of proportion of fusion genes between high and low-risk score subgroup

| **Fusion genes** | **Total** | **Low-risk score (93)** | **High-risk score (271)** | ***p*** |
| --- | --- | --- | --- | --- |
| Complex | 2(0.7%) | 2(2.2%) | 0(0.0%) | 0.065 |
| *CBFB*-*MYH11* | 22(6.4%) | 1(1.1%) | 21(7.7%) | 0.038 |
| *DEK*-*NUP214* | 3(0.7%) | 0(0.0%) | 3(1.1%) | 0.573 |
| *GATA2*-*MECOM* | 7(2.0%) | 0(0.0%) | 7(0.4%) | 0.198 |
| *MLLT3*-*KMT2A* | 11(3.2%) | 10(10.8%) | 1(0.0%) | < 0.001 |
| *PML*-*RARA* | 12(3.7%) | 4(4.3%) | 8(3.0%) | 0.770 |
| *RUNX1*-*RUNX1T1* | 11(3.0%) | 2(2.2%) | 9(3.3%) | 0.827 |

*p* < 0.05 is considered statistically significant.

Thirty nine patients with unknown fusion genes data were excluded in the chi-square test.

**Table S4** Comparison of treatment types between patients with high and low-risk score

| **Treatment types** | **Yes or No** | **High-risk score**  **（286）** | **Low-risk score**  **（103）** | ***p*** |
| --- | --- | --- | --- | --- |
| Chemotherapy | Yes | 276 (96.5%) | 98 (95.1%) | 0.539 |
|  | No | 10 (3.5%) | 5(4.9%) |  |
| Transplant | Yes | 77 (26.9%) | 35 (34.0%) | 0.175 |
|  | No | 209 (73.1%) | 68 (66.0%) |  |
| Targeted therapy* | Yes | 61 (21.3%) | 10 (9.7%) | 0.009 |
|  | No | 225 (78.7%) | 93 (90.3%) |  |

*p* < 0.05 is considered statistically significant.

14 patients who lacked treatment data were not included in this chi-square test.

Specific types of targeted therapy were unavailable.

**Table S5** Univariate analysis of overall survival in TCGA cohort

| **Variables** | **HR** | **95% confidence interval** | | ***p*** |
| --- | --- | --- | --- | --- |
|  |  | **Lower limit** | **Upper limit** |  |
| Elderley (> 60 years old) | 3.132 | 2.134 | 4.599 | < 0.001 |
| WBC count* | 1.005 | 1.001 | 1.009 | 0.016 |
| HSCT | 0.524 | 0.357 | 0.7685 | < 0.001 |
| Complex karyotype | 1.883 | 1.372 | 2.439 | 0.017 |
| Blasts in bone marrow * | 1.000 | 0.990 | 1.010 | 0.996 |

*p* < 0.1 is considered statistically significant.

HR, hazard ratio; WBC, white blood cell; HSCT, hematopoietic stem cell transplantation.

*Continuous variable.

**Table S6** Spearman correlation analysis of *CALCRL* expression with *ABCB1*, *ABCC1*, and *ABCG2*

|  | ***ABCB1*** | ***ABCC1*** | ***ABCG2*** |
| --- | --- | --- | --- |
| correlation coefficient | 0.271 | 0.461 | 0.117 |
| *p* | < 0.001 | < 0.001 | 0.013 |

*p* < 0.05 is considered statistically significant.

**Table S7** Multivariate Cox regression analysis of the risk score and the *CBFB-MYH11* fusion gene

|  | **coef** | **exp(coef)** | **se(coef)** | **z** | **Pr(>\|z\|)** | **exp(-coef)** | **lower .95** | **upper .95** |
| --- | --- | --- | --- | --- | --- | --- | --- | --- |
| risk.scoreLow | -0.3726 | 0.6889 | 0.1702 | -2.189 | 0.0286 | 1.452 | 0.4935 | 0.9617 |
| *CBFB-MYH11*(+) | -0.9885 | 0.3721 | 0.4155 | -2.379 | 0.0173 | 2.687 | 0.1648 | 0.8401 |

coef: coefficients, beta value.

exp(coef): hazard ratio.

se(coef): standard error.

z: Wald value.

Pr(>|z|): p value.

**
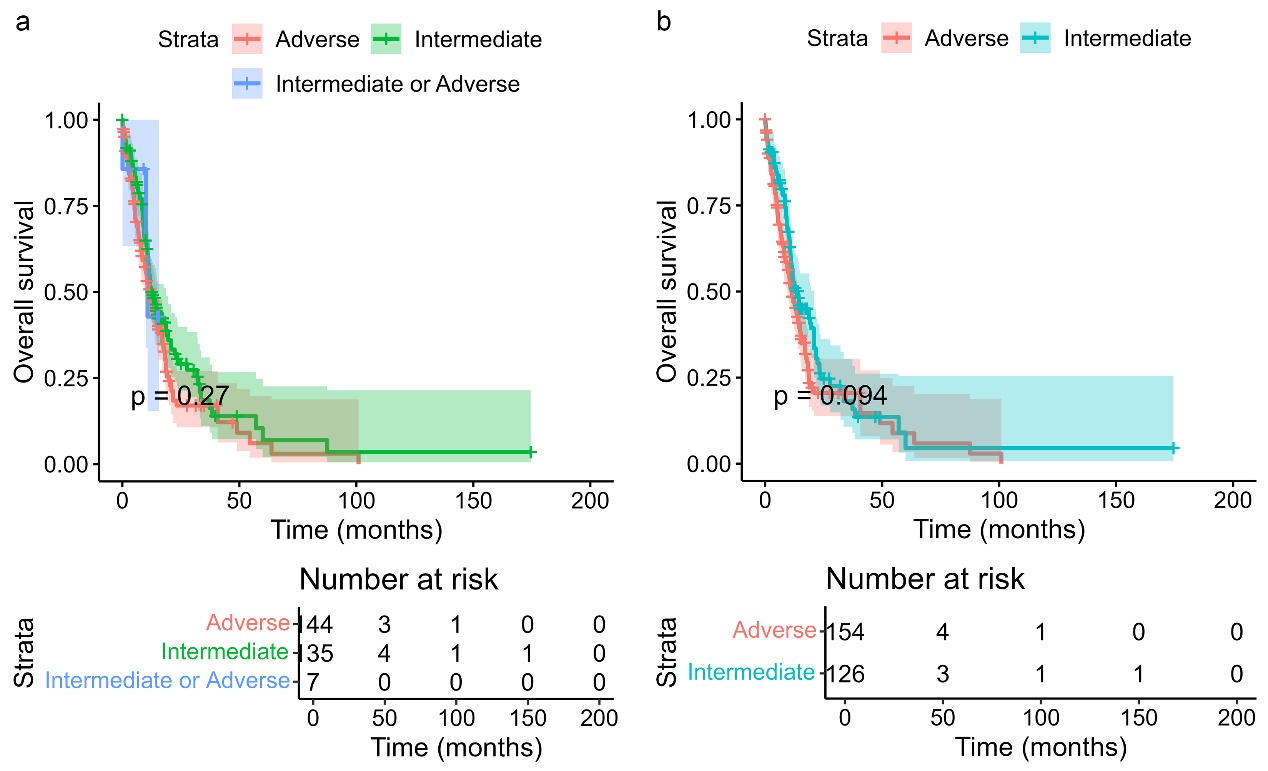
**

**Figure S1** Kaplan-Meier Analysis of Overall Survival (OS) of non-favorable-risk groups in OHSU cohort. (a) OS of patients classified as “intermediate” or “intermediate or adverse” or “adverse” risk according to the ELN2017 guidelines. (b) OS of patients classified as “intermediate” or “adverse” risk according to the ELN2022 guidelines. The analysis was performed after excluding patients in the favorable risk group. No significant difference was observed.


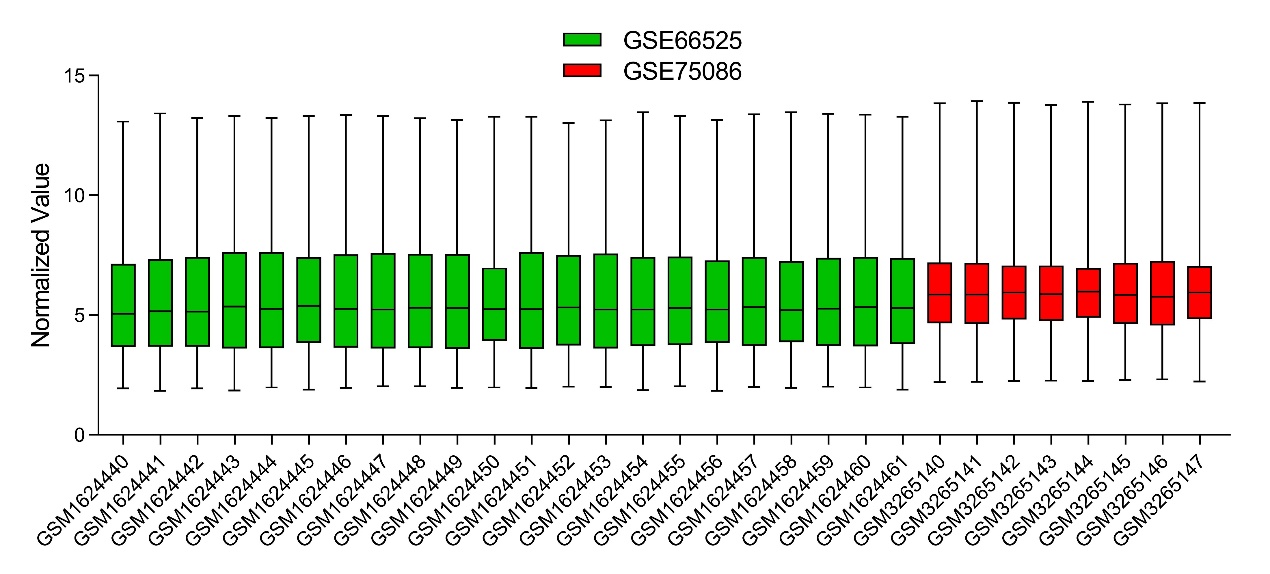


**Figure S2** Boxplot of the log2-transformed gene expression value measured by microarray in each sample. Green color represents GSE66525, red color represents GSE75086. The median normalized gene expression values of different samples were concentrated at the same level, indicating that the differences were relatively small.


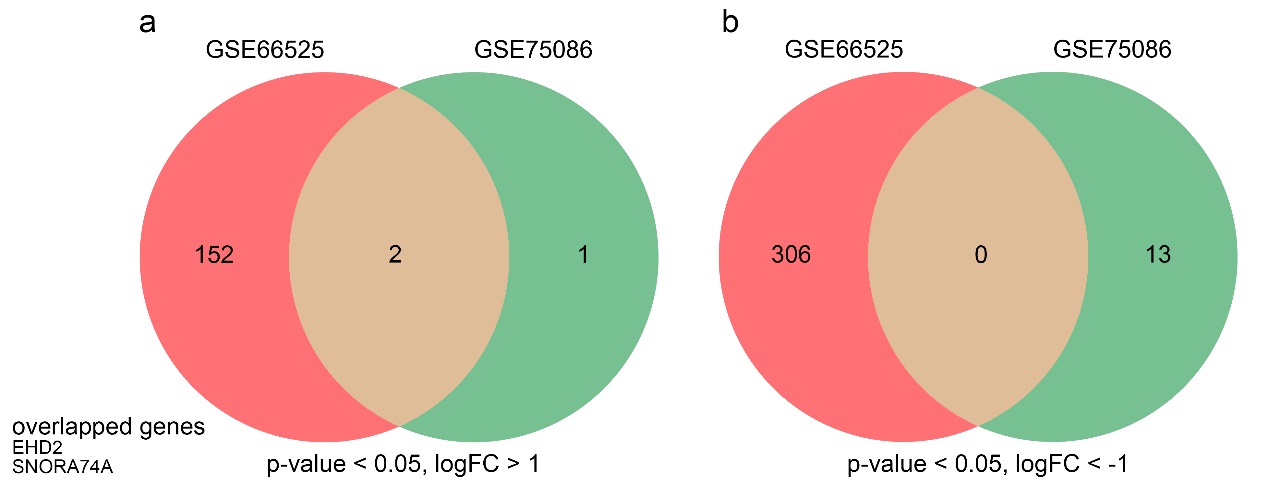


**Figure S3** The Venn diagram of overlapping DEGs identified from the two datasets. (a) upregulated genes (*p* < 0.05, logFC > 1). Two genes *EHD2* and *SNORA74A* are upregulated in both datasets. (b) downregulated genes (*p* < 0.05, logFC < -1). No overlapping downregulated genes were found.
